# Supplementary material for: Lipid Membrane State Change by Catalytic Protonation and the Implications for Synaptic Transmission
Source: Membranes (Basel). 2021 Dec 21;12(1):5. doi: 10.3390/membranes12010005 (PMC8781637; doi:10.3390/membranes12010005)
Supplement: Supplementary file 1 [file membranes-12-00005-s001.zip › membranes-1430029-supplementary.pdf]

Supplementary Materials: Lipid membrane state change by catalytic protonation and the implications for synaptic transmission

Christian Fillafer [1]*, Yana S. Koll [1] and Matthias F. Schneider [1]

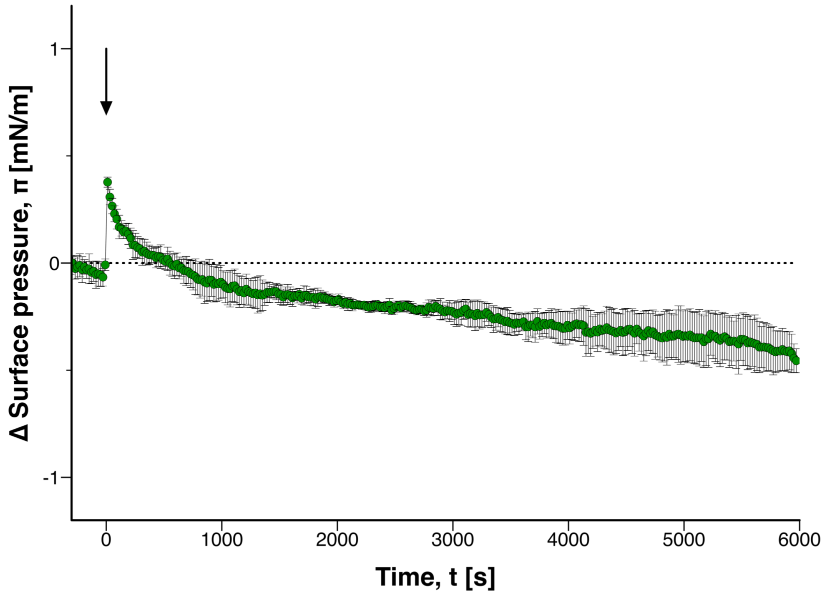

Figure S1. Surface pressure change upon injection of $V = 160\,\mu L$ of subphase onto DMPS monolayer. The initial surface pressure after equilibration for 60 min at 23°C was $\pi \approx 20\,mN/m$. The data series consists of mean values StDev of $n = 3$ measurements.

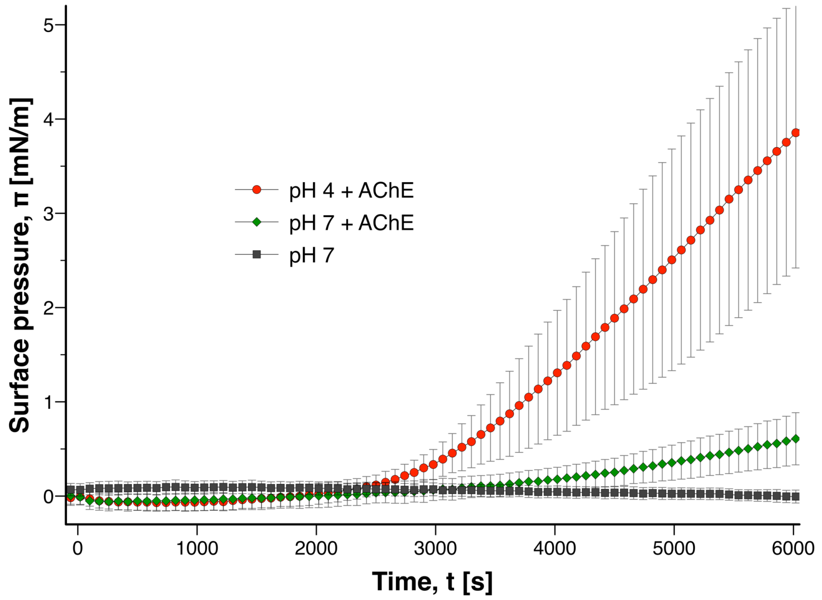

Figure S2. pH-dependent surface activity of acetylcholinesterase (AChE) from *E. electricus*. Change of surface pressure of the gas-solution interface when AChE was added to standard subphase at pH 4 *(red circles)* or pH 7 *(green diamonds)*. Control experiments: change of π without AChE at pH 7 *(black squares)*. Each data series consists of mean values StDev of $n = 2 - 3$ measurements.

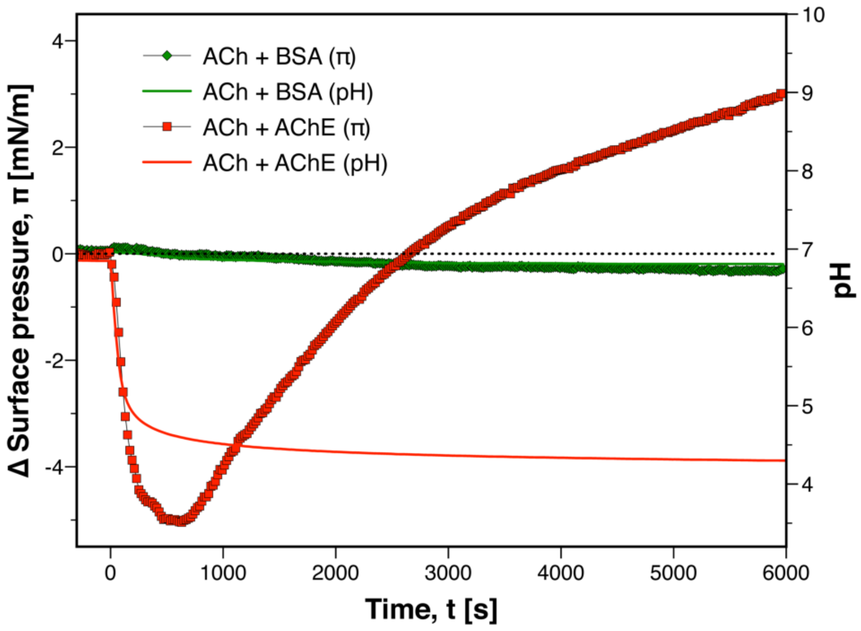

Figure S3. Injection of ACh onto a DMPS monolayer in presence of bovine serum albumin (BSA). Changes of surface pressure π (left axis, symbols) and subphase pH (right axis, solid lines) when ACh was injected at $t = 0$. The subphase contained either AChE (red) or BSA (green). The data for BSA are a representative example taken from a series of $n = 2$ measurements. The data for AChE were replotted from Fig. 2 in the main text for comparison.

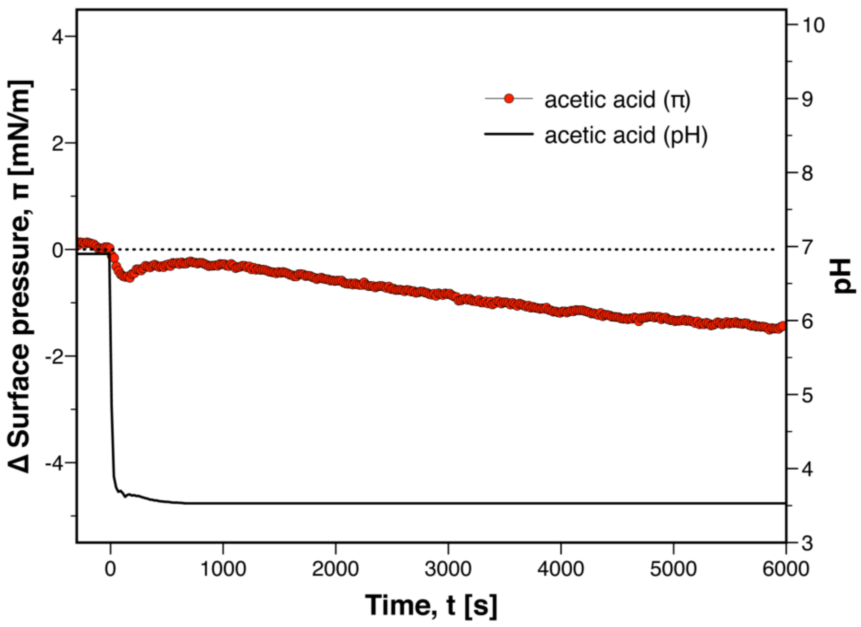

Figure S4. Response of a monolayer made from DPPC to injection of acetic acid. Changes of surface pressure π (left axis, symbols) and subphase pH (right axis, solid line) when acetic acid was injected at $t = 0$. The final concentration of acid was $c = 10mM$. Data series is a representative example taken from a series of $n = 2$ measurements.

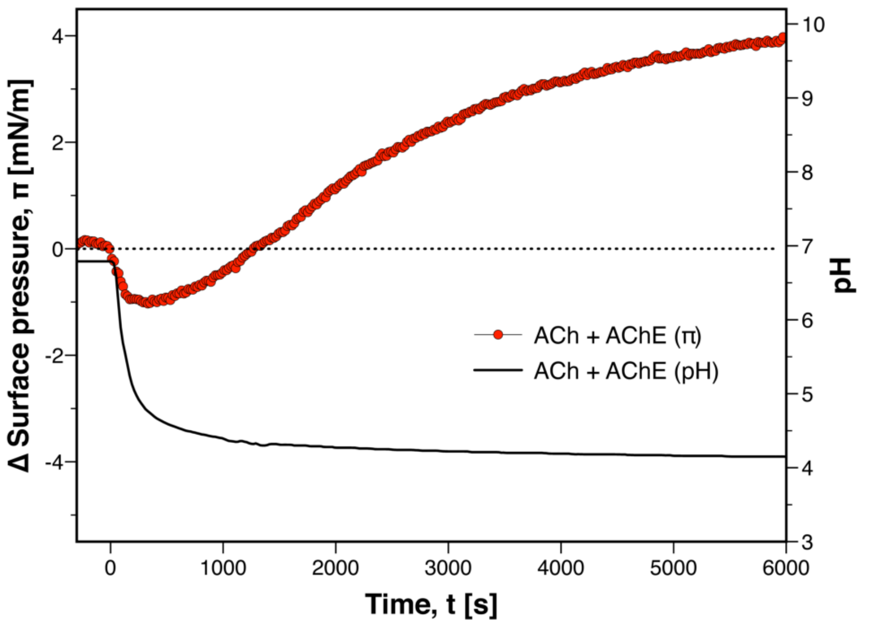

Figure S5. Response of a monolayer made from DPPC to injection of ACh in presence of AChE. Changes of surface pressure π (left axis, symbols) and subphase pH (right axis, solid line) when ACh was injected at $t = 0$. The final concentration of ACh was $c = 10mM$. Data series is a representative example taken from a series of $n = 2$ measurements.

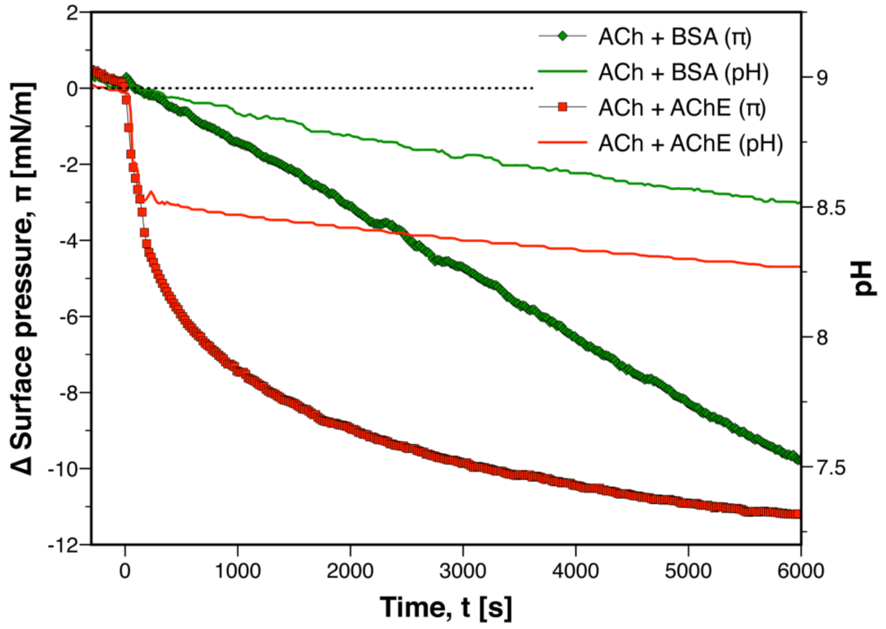

Figure S6. Injection of ACh onto a DMPA monolayer in presence of bovine serum albumin (BSA). Changes of surface pressure π (left axis, symbols) and subphase pH (right axis, solid lines) when ACh was injected at $t = 0$. The subphase contained either AChE (red) or BSA (green). The data for BSA are a representative example taken from a series of $n = 2$ measurements. The data for AChE were replotted from Fig. 4 in the main text for comparison.
